# National Conference of State Boards of Health

**Published:** 1895-11

**Authors:** 


At the National Conference of State Boards of Health, held at Washington, D. C., December 13, 1894, the following resolution was introduced by this Board and unanimously adopted:

WHEREAS, The increasing pollution of bodies of water contiguous to cities and towns has become a menace to the health of communities of such gravity and extent that it is a question of National importance; therefore, be it

*Resolved*, That it the sense of this Conference that the whole subject of the contamination of such lakes and streams as are the source of water supply to more than one State, should be investigated by a commission created by act of Congress, and that the conclusions reached, together with suggestions for legal remedy and control, should be published from time to time for the information of interested communities.

A bill to create a commission with duties in the line of this expression of a National Sanitary need was introduced in the house (H.R., 8481) January 12, 1895, by the Hon. Richard Bertholdt, of Missouri.

